# Effects of Nanoparticle Size on the Thermal Decomposition Mechanisms of 3,5-Diamino-6-hydroxy-2-oxide-4-nitropyrimidone through ReaxFF Large-Scale Molecular Dynamics Simulations

**DOI:** 10.3390/molecules29010056

**Published:** 2023-12-21

**Authors:** Zijian Sun, Jincheng Ji, Weihua Zhu

**Affiliations:** 1 Institute for Computation in Molecular and Materials Science, School of Chemistry and Chemical Engineering, Nanjing University of Science and Technology, Nanjing 210094, China; zijian.sun@njust.edu.cn; 2 College of Chemical Engineering and Pharmacy, Jingchu University of Technology, Jingmen 448000, China; forbiddenciy_jjc@163.com

**Keywords:** reactive molecular dynamics, IHEM−1 nanoparticles, decomposition mechanisms, high temperatures, reaction kinetics

## Abstract

ReaxFF-lg molecular dynamics method was employed to simulate the decomposition processes of IHEM−1 nanoparticles at high temperatures. The findings indicate that the initial decomposition paths of the nanoparticles with different sizes at varying temperatures are similar, where the bimolecular polymerization reaction occurred first. Particle size has little effect on the initial decomposition pathway, whereas there are differences in the numbers of the species during the decomposition and their evolution trends. The formation of the hydroxyl radicals is the dominant decomposition mechanism with the highest reaction frequency. The degradation rate of the IHEM−1 molecules gradually increases with the increasing temperature. The IHEM−1 nanoparticles with smaller sizes exhibit greater decomposition rate constants. The activation energies for the decomposition are lower than the reported experimental values of bulk explosives, which suggests a higher sensitivity.

## 1. Introduction

The design and application of insensitive energetic compounds are essential in the fields of defence and civil industry, particularly in nuclear weapons and space exploration, due to the unique application environment. 3,5-Diamino-6-hydroxy-2-oxide-4-nitropyrimidone (IHEM−1) [1] is an ideal compound since it has numerous advantages, including its simple preparation, high yield, high density, low solubility in aqueous solution, and good detonation properties. Considering its comparable safety and synthetic feasibility with 1,3,5-triamino-2,4,6-trinitrobenzene (TATB), as well as its higher energy density, IHEM−1 could be a viable alternative to TATB [1]. Hence, obtaining a credible description of its thermal decomposition kinetics becomes crucial to preventing or controlling the explosion of explosives. Additionally, this research will aid in comprehending the ignition, combustion, and detonation mechanisms [2].

The rapid reaction process of the explosives involves short time scales and complex chemical reaction kinetics. Moreover, the reaction usually occurs under extreme conditions. It proves difficult for existing experimental techniques and traditional simulation methods to ascertain the detailed reaction mechanisms. Hence, this poses a significant challenge to the application and development of the explosives. In previous decades, some researchers experimentally investigated the thermal decomposition properties of the explosives. For example, Khichar et al. [3] investigated the detailed thermal decomposition mechanisms of liquid-phase RDX via FTIR spectrometry, and the results showed that their proposed liquid-phase decomposition mechanisms of RDX-based single-component propellant are applicable to the operating pressure of rocket motors. In recent years, the computational method has become a powerful tool to simulate the decomposition of the explosives. Based on the density-functional tight-binding molecular dynamics (DFTB-MD) method with a multiscale shock technique (MSST), Zhang [4] studied the decomposition mechanisms and hydrogen transfer process of CL-20/TNT cocrystal under shock loading. The results indicated that the increasing volume reduction led to higher temperature and pressure when the system suffered from a stronger shock strength. Zhang et al. [5] simulated the pyrolysis mechanism of RDX via ab initio kinetic calculations. The simulation results show that the decomposition of RDX is mainly through the cleavage of the N-NO_2_ bond to form RDXR.

The computational complexity of ab initio calculations limits its application to large-scale systems despite the fact that its computational accuracy is relatively high. However, recently, reactive molecular dynamics (RMD) has emerged as a practical approach to simulate the reaction processes of explosives under extreme circumstances on a large scale. Reactive Force Field Molecular Dynamics (ReaxFF-MD) simulations are widely used for evaluating reactivity [6]. This technique enables direct observation of the evolution of chemical reactions at a microscopic level, achieving atomic-level insight at relevant spatial and temporal scales for researchers [7,8,9]. RMD has been successfully used to model the thermal decomposition reactions of 1,3,5-trinitroperhydro-1,3,5-triazine (RDX) [10], 1,3,5 hexanitrohexaazoisovuttzane (CL-20) [11], 1,3,5,7-tetranitro-1,3,5,7-tetrazocine (HMX) [12], TATB [13], pentaerythritol tetranitrate (PETN) [14], dihydroxylammonium 5,5′-bistetrazole-1,1′-diolate (TKX-50) [15], and so on. Furthermore, Wang et al. [16] have developed the ReaxFF reactive force field for hydrocarbons and have studied the thermal decomposition of isooctane. More recently, Sultan et al. [17] investigated the thermal decomposition mechanism of CL-20/DNT cocrystal at different high temperatures using ReaxFF-MD simulations. These successes show that the ReaxFF reactive force field can not only afford the computational cost of studying large models but also maintain near-quantum-mechanical accuracy. Liu et al. [18] subsequently refined the ReaxFF force field by addressing long-range interactions to obtain an improved ReaxFF-lg force field, which provides crystal densities that are more close to the experimental data. Recently, numerous researchers have conducted extensive studies on the decomposition of explosives in various states by utilizing RMD with an improved ReaxFF-lg force field [19,20,21,22,23,24].

Understanding the decomposition mechanisms of explosives under extreme conditions is crucial for the design of new high explosives [25]. Before the decomposition, the explosives will undergo changes in molecular and crystal structures and phase transition [6,26,27,28,29,30]. The decomposition mechanisms of the explosives are often affected by molecular stacking patterns, crystal morphology, particle sizes, defects, etc.; therefore, it is very important in terms of the effects of these factors on the decomposition process. Recently, ReaxFF/lg force field MD was used to investigate the effects of particle sizes, crystalline phases, and defects on the decomposition of RDX [31,32,33], nitromethane (NM) [34], 2,2′,4,4′,6,6′-hexanitrostilbene (HNS) [35], composition B melt-cast explosive [36], plastic-bonded explosives (PBXs) [37], and nano ε-CL-20 [38]. These studies have demonstrated the dependability and efficacy of ReaxFF-lg MD in studying the thermal decomposition of energetic materials.

Nanomaterials have attracted much attention in the field of explosives due to their special properties. They have many features such as small size effect, surface effect, quantum size effect, and macroscopic quantum tunnelling effect, which makes them possess unique properties. In order to develop new energetic materials, an in-depth study on the behaviors of nano explosives is needed.

In this work, the thermal decomposition of nano IHEM−1 particles at different temperatures from 2000 to 3500 K was simulated using RMD with the ReaxFF-lg force field. This study explores the initial decomposition pathways, the evolution of intermediates and final products, and reaction kinetics. Additionally, the effects of nanoparticle size on thermal decomposition were examined. This work may contribute to establishing a theoretical comprehension of the decomposition process of nano-explosives.

## 2. Results and Discussion

### 2.1. Early Evolution of the IHEM−1 Molecules

**Figure 1:**
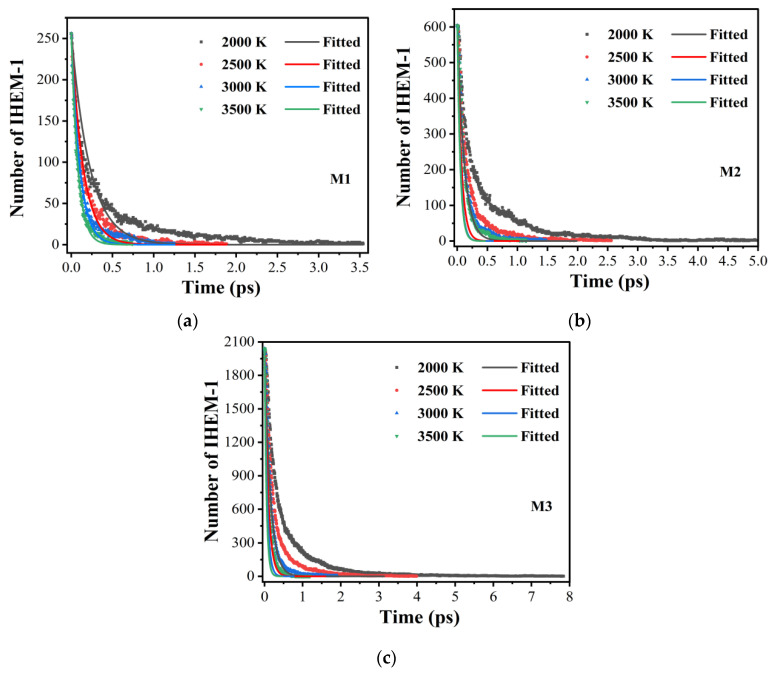
Evolution of the numbers of the IHEM−1 molecules in the M1 (**a**), M2 (**b**), and M3 (**c**) systems during the initial decomposition stages of the three models at different temperatures.

Figure 1 depicts the numbers of the IHEM−1 molecules during the initial decomposition stage for the three IHEM−1 nanoparticles at high temperatures with time. As seen in Figure 1a, it is apparent that the reactants decayed to zero in 3.54, 1.885, 1.255, and 0.745 ps for M1 at 2000, 2500, 3000, and 3500 K, respectively. Similarly, Figure 1b shows that the reactants decayed to zero in 4.965, 2.56, 1.485, and 1.15 ps for M2 at the four reaction temperatures, respectively. Likewise, the reactants for M3 decayed to zero in 7.785, 3.765, 1.915, and 1.195 ps at four varying reaction temperatures, respectively. These findings show that the decomposition of the reactants becomes faster and faster as the reaction temperature increases.

Table 1 outlines the time taken for the reactants to decompose to zero for the three models at varying temperatures. As the particle size increases, the decay time increases in the order of M1, M2, and M3. The decay rate of IHEM−1 significantly increases with the increasing temperature. This suggests that the reactivity of IHEM−1 is higher at higher temperatures. In addition, smaller IHEM−1 nanoparticles display higher decay rates during decomposition. These findings correspond with the nanocrystal size effects in RDX established in Zheng’s theoretical studies [39]. Furthermore, Huang’s experiments suggest that a reduction in the average particle size of nano-FOX-7 leads to a very high rate of decomposition [40]. Additionally, our results are in line with the nano size effects in other inorganic nanomaterials [41,42,43].

### 2.2. Evolution of Total Species

**Figure 2:**
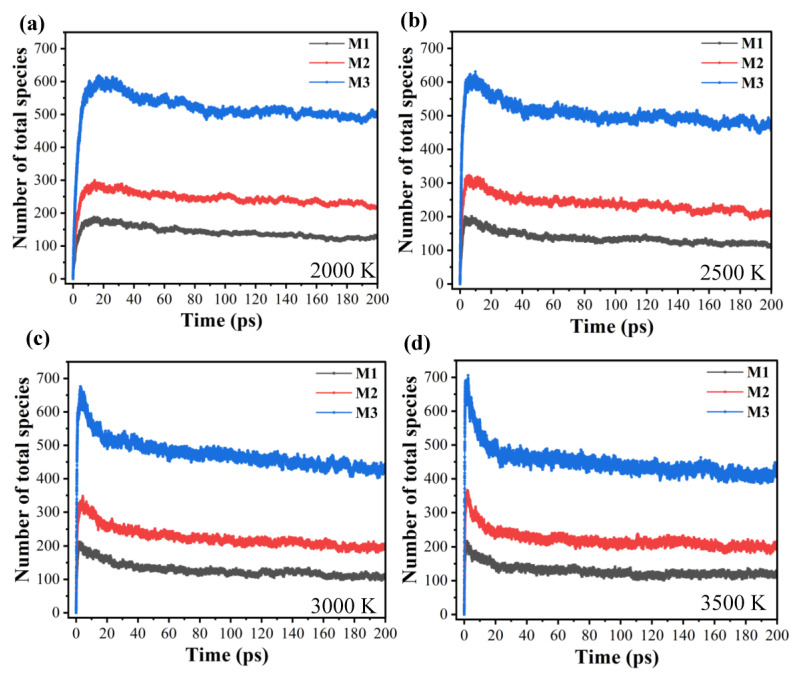
Evolution of the numbers of the total species in the decomposition for the three models at 2000 K (**a**), 2500 K (**b**), 3000 K (**c**), and 3500 K (**d**).

Figure 2 illustrates the evolution of the total species in the decomposition of the three nanoparticles of IHEM−1 at 2000, 2500, 3000, and 3500 K. As illustrated in Figure 2, the total quantity of three species within M1, M2, and M3 increases with the increasing temperature. When the temperature is fixed, increasing nanoparticle diameter leads to an increase in the total number of species in the system. This trend is evident. Additionally, the higher the temperature is, the shorter the time required for total numbers of species to peak. The influence of the nanoparticle’s diameter on the total quantity of the species is more pronounced within the system in comparison to the temperature. The findings indicate that enhancing temperature expedites the breakdown of the IHEM−1 molecules, resulting in an increase in the quantity of products. It is worth noting that at the point where the overall number of the species in the system reaches a maximum, the numbers of the species in the system experience a small decline prior to reaching a specific value. This pattern indicates that the overall numbers of the species in the system also maintain a stable state after reaching equilibrium.

### 2.3. Initial Decomposition Pathway

To investigate the thermal decomposition mechanisms of the IHEM−1 molecules, we analyzed the trajectory files of M1-M3 using the post-processor developed by Zeng [44]. It is found that the initial decomposition pathways for the three systems are similar at different temperatures, and the differences lie in the numbers of the species produced during decomposition and their evolution trend. The sizes of the IHEM−1 nanoparticles have no effect on the initial decomposition pathway. Therefore, we chose M3 as an illustration to analyze its first 10 ps trajectory file at 3000 K. The simulation results reveal that there are a total of 17 potential decomposition channels during the initial decomposition of M3, as detailed in Table 2. They include intramolecular hydrogen transfer (paths 1 and 2), intermolecular hydrogen transfer (paths 3 and 4), the formation of hydroxyl radicals (path 5), the formation of amino radicals (paths 6 and 8), the formation of nitro radicals (path 7), the formation of hydroxyl radicals (paths 9 and 10), the formation of water molecules (path 11), carbon–nitrogen bond breaking in the ring (paths 12–15), rearrangement of NO_2_ groups (path 16), and bimolecular polymerization reaction (path 17).

The primary reaction paths were deduced based on the ratios and frequencies of the principal reaction products. There exist five primary reaction mechanisms during the thermal decomposition of M3: The first path is the bimolecular polymerization reaction of the IHEM−1 molecule (Mechanism 1: C_4_N_5_O_5_H_5_ + C_4_N_5_O_5_H_5_→C_8_N_10_O_10_H_10_). The next one is the formation of hydroxyl radicals (Mechanism 2: C_4_N_5_O_5_H_5_→C_4_N_5_O_4_H_4_ + OH). The following one is a hydrogen transfer (Mechanism 3: H + NO_2_→HNO_2_), a process that produces the third and fourth occurrence of major intermediates, C_4_N_5_O_5_H_4_ and C_4_N_5_O_5_H_6_. The C-NO_2_ bond undergoes homogeneous breakage, subsequently releasing NO_2_, named Mechanism 4 (C_4_N_5_O_5_H_5_→C_4_N_4_O_3_H_5_ + NO_2_). Finally, Mechanism 5 involves the dissociation of the C-N bond in the ring. The occurrence frequency of the five primary decomposition pathways of IHEM−1 is in the order of Mechanism 1 > Mechanism 2 > Mechanism 3 > Mechanism 4 > Mechanism 5. Next, throughout the decomposition of IHEM−1, the occurrence frequency of the main decomposition products over time will be analyzed to detail the thermal decomposition mechanisms.

### 2.4. Evolution of Main Intermediates

**Figure 3:**
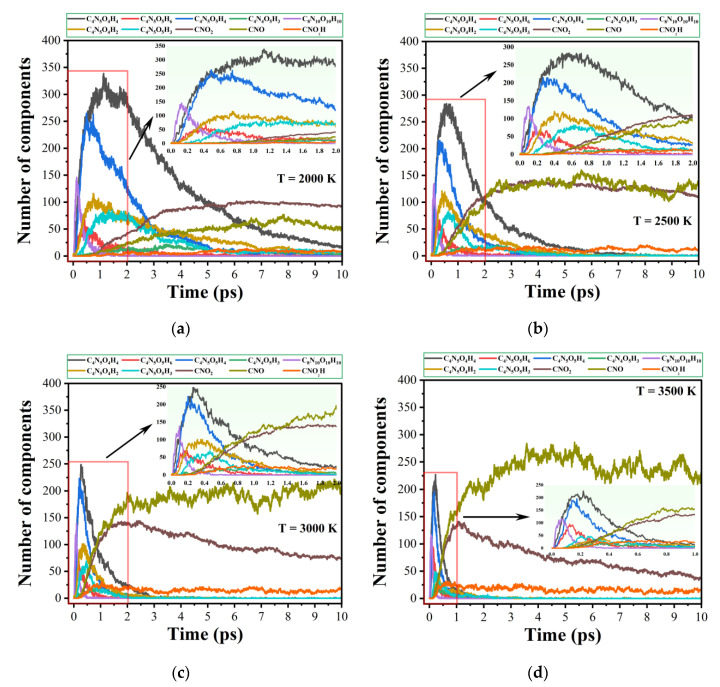
Evolution of the numbers of the main intermediates in the decomposition of Model 3 at 2000 K (**a**), 2500 K (**b**), 3000 K (**c**), and 3500 K (**d**).

Figure 3 illustrates the evolution of the numbers of the major intermediate and secondary products during the thermal decomposition of M3. It is found that the appearance of C_4_N_4_O_5_H_3_ (C_4_N_5_O_5_H_5_→C_4_N_4_O_5_H_3_ + NH_2_) is late and its yield is low, suggesting that the formation of the NH_2_ group is not the main decomposition mechanism. The primary products C_4_N_5_O_4_H_4_ and C_4_N_5_O_5_H_4_ will further decompose to produce secondary products C_4_N_5_O_4_H_2_ and C_4_N_5_O_5_H_3_. Meanwhile, by analyzing the trajectory files, it is known that the main intermediates produced by the C-N bond breaking in the ring are CNO_2_, CNO, and CNO_2_H, and their related reactions are C_4_N_5_O_4_H_2_→C_3_N_4_O_3_H_2_ + CNO, C_4_N_5_O_5_H_3_→C_3_N_4_O_3_H_3_ + CNO_2_, and C_4_N_5_O_5_H_5_→C_3_N_4_O_3_H_4_ + CNO_2_H.

The peak values of the numbers of CNO_2_, CNO and CNO_2_H become increasingly large with increasing temperature. The earliest pathway for the formation of water is through the H atom of the amino group in the IHEM−1 molecule attracted by the O atom of the hydroxyl group. NO_2_ comes mainly from the breaking of the C-NO_2_ of IHEM−1, and NO comes mainly from the rearrangement of the NO_2_ group and the further decomposition of the intermediates (-CNO and -CNO_2_). For most nitro explosives, the frequency of C-NO_2_ cleavage is higher than that of the -NO_2_ rearrangement [45], consistent with the conclusions drawn from our simulations. The succeeding primary decomposition process involves the reactions between small molecule products.

### 2.5. Evolution of Small Molecule Products

**Figure 4:**
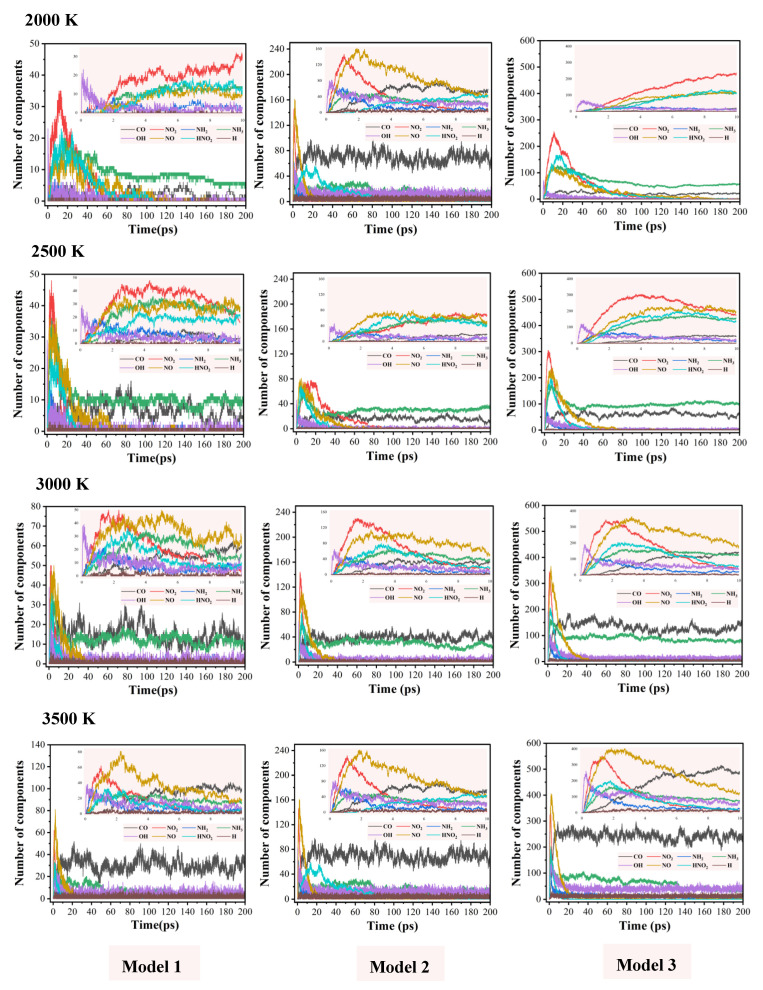
Evolution of the numbers of small molecule products during the decomposition of the three models at different temperatures.

Figure 4 shows the evolution of the number of small molecule products produced during the thermal decomposition of the three models. The small molecule products are CO, NO_2_, and NO, and the free radicals NH_2_, NH_3_, OH, HNO_2_, and H. It can be seen in Figure 4 that the size of the nanoparticle significantly influences the evolution of the number of NH_3_, whereas the temperature has a minor effect. Additionally, the amount of CO increases as both the temperature and particle size increase. The amount of hydrogen radicals is the least, which were formed by the N-H dissociation of the amino group moiety. The hydrogen radicals continued to be involved in the reaction of paths 1–4 (H + NO_2_→HNO_2_, O + H→OH), where HNO_2_ will further decompose to produce OH and NO. Therefore, the evolution trend of the number of HNO_2_ in Figure 4 is consistent with that of small radicals OH, NH_2_, NO, and NO_2_, which show a trend of increasing to a peak and then decreasing.

In all three systems, OH is the first decomposition product to appear. The nanoparticles of IHEM−1 with larger diameters generate a larger number of OH at higher temperatures. OH originates from two paths: one is the decomposition of the HNO_2_ radical, and the other is produced by the reaction O + H→OH, which has a large change in the number depending on the temperature and diameter of the nanoparticle. Specifically, at 3500 K, the amount of OH in M1, M2, and M3 at the equilibrium are around 10, 20, and 50, respectively.

**Figure 5:**
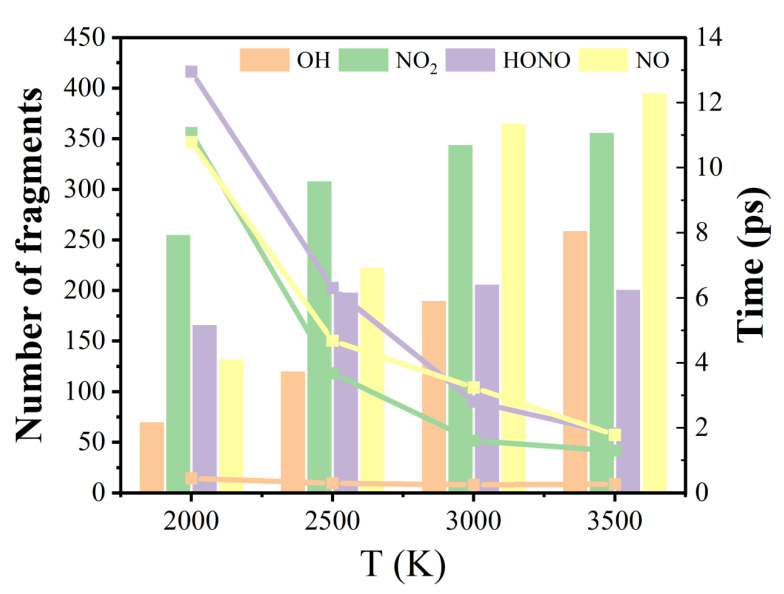
Peak values (column chart) and corresponding occurrence time (line chart) for the numbers of OH, NO_2_, HONO, and NO in the decomposition of M3 at different temperatures.

Figure 5 illustrates the peak values (bar graphs) and corresponding times (line graphs) of the major products OH, NO_2_, HONO, and NO during the thermal decomposition of M3. The peak values of the number of NO_2_ are larger than those of NO when the temperatures are 2000 and 2500 K, whereas the cases are the opposite at 3000 and 3500 K. This may be because there are three production pathways of NO, one is the decomposition of HNO_2_, the second is through the rearrangement of -NO_2_, and the third is through the further reaction of -CNO. NO_2_ mainly comes from the C-NO_2_ cleavage of the IHEM−1 molecules. Since high temperature promotes the decomposition of the third pathway, more NO was produced at higher temperatures.

As the temperature increases, the time required for the number of the major products OH, NO_2_, HONO, and NO to reach their peak values gradually decreases, but their peak values become more and more large. It means that the temperature can accelerate the decomposition of the reactants and thus promote the formation of the intermediates. The peak value of the number of HNO_2_ at 3500 K is slightly smaller than that at 3000 K. It may be speculated that the C-NO_2_ bond is easier to break at 3500 K, in agreement with previous report that higher temperatures are more conducive to the production of NO_2_ [46], which reduces the possibility of hydrogen transfer.

### 2.6. Evolution of Final Products

**Figure 6:**
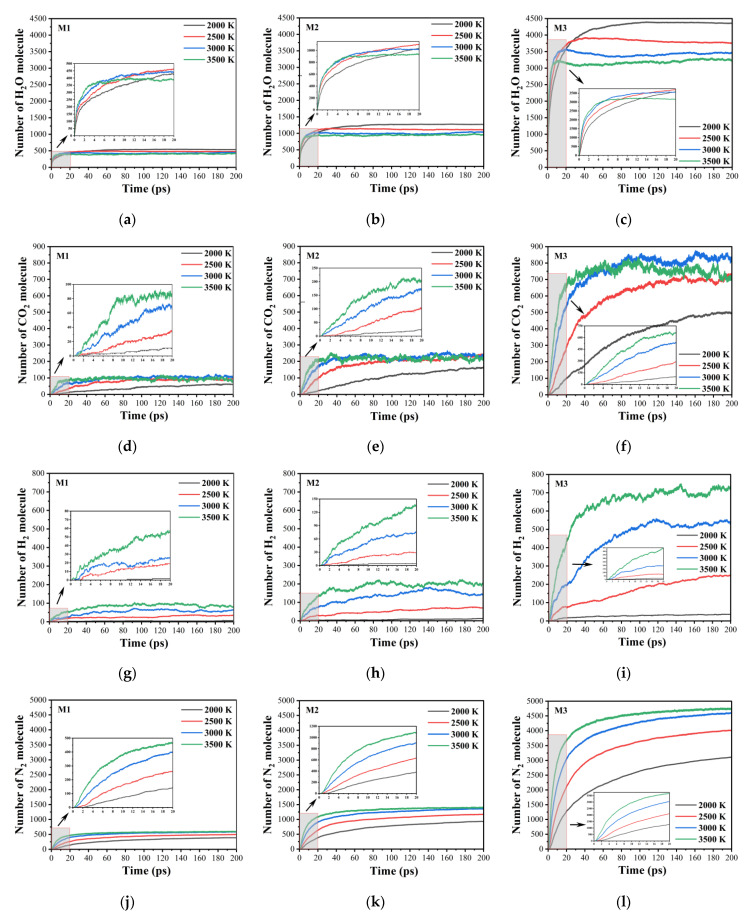
Evolution of the numbers of the final products H_2_O (**a**–**c**), CO_2_ (**d**–**f**), H_2_ (**g**–**i**), and N_2_ (**j**–**l**) in the decomposition for the three models at different temperatures.

Figure 6 shows the evolution of the numbers of the final products H_2_O, CO_2_, H_2_ and N_2_ during the decomposition of the three models at different temperatures. The evolution trends of the numbers of the four final products are consistent: their amounts increase first, then reach their peaks, and finally attain their equilibrium. The amount of H_2_O, CO_2_, H_2_ and N_2_ in the three systems is in the order of M3 > M2 > M1. Therefore, the larger the nanoparticle’s size is, the larger the numbers of the final products in the system are. As seen in Figure 6a–c, the numbers of H_2_O in the system increase with the increasing temperature at the early decomposition stage (about before 4.5 ps), but after 4.5 ps, they decrease gradually at 3500 K. This indicates that higher temperatures inhibit the production of H_2_O. A possible reason is that high temperature promotes the participation of H_2_O as a reactant in more secondary and intermediate reactions, leading to a slight decrease in the number and eventually reaching an equilibrium. In addition, the higher the temperature is, the greater the promotion effect is. As shown in Figure 6d–f, during 0–40 ps, the amount of CO_2_ increases with increasing temperature. Afterwards, this evolution trend no longer exists at 3000 and 3500 K. The possible reason is that the effects of the temperature on the total number of species at the equilibrium stage are very small.

The amount of H_2_O, CO_2_, H_2_, and N_2_ in the three models are in the sequence of N_2_ > H_2_O > CO_2_ > H_2_. The amount of H_2_ is smaller compared to CO_2_, H_2_O, and N_2_. The formation rates of H_2_ are similar for the three systems (Figure 6g–i). At different temperatures, the formation rates of N_2_ in the three systems are in the order of M3 > M2 > M1 (Figure 6j–l). High temperatures have a significant effect on their formation, and the higher the temperature is, the greater the promotion is. Among all the final products, the number of N_2_ is the largest, mainly from the secondary reaction of nitrogen dioxide and NO, and its formation mechanism is the reaction of nitrogen dioxide or NO with carbon-containing fragments to produce N_2_, consistent with the results achieved in previous studies [25].

### 2.7. Decomposition Reaction Kinetics

In order to further investigate the effects of temperature and particle size on the initial decomposition, we quantitatively measured the instantaneous decomposition rate constants of the IHEM−1 nanoparticles with different sizes at different temperatures. The decay rate constant *k* can be calculated via the first-order expression (1) [35].

*N*(*t*) = *N*_0_ · exp[ − *k*(*t* − *t*_0_)] (1)

where *N*_0_ is the initial amount of reactants, *t*_0_ is the onset time of initial decomposition, and *k* is the initial reaction rate. It can be seen in Table 3 that the decomposition rate of the IHEM−1 nanoparticles gradually accelerates with the increasing temperature during the initial decomposition stage. Therefore, an increase in the temperature accelerates the initial decomposition of the reactants. At the same temperature, it is observed that the system with smaller particle’s size has a higher rate constant (*k*_M1_ > *k*_M2_ > *k*_M3_). This implies that the kinetic decay rate of the IHEM−1 nanoparticles with smaller size is faster.

**Figure 7:**
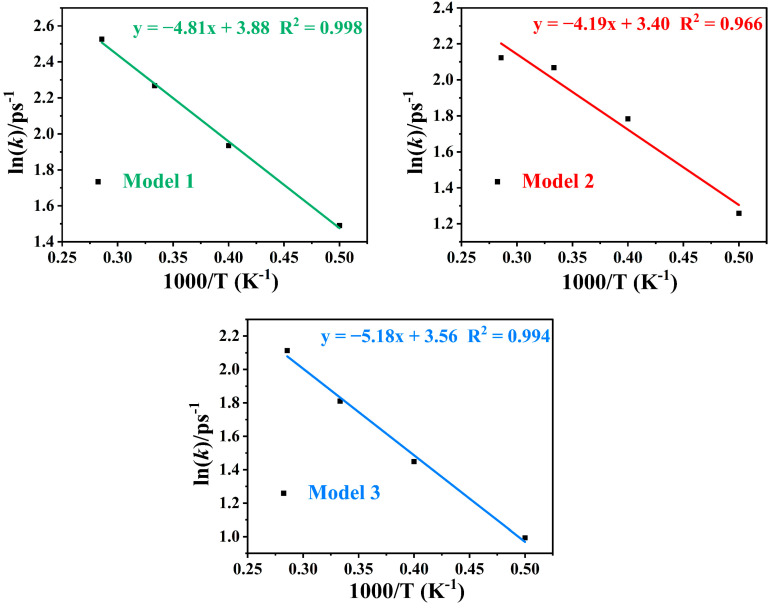
Logarithm of the initial decomposition rate (ln*k*) against the inverse temperature (1/T) for the three models at different temperatures.

In order to obtain the pre-exponential factor and activation energy of the decomposition reaction, we fitted linear Equation (2).

ln(*k*) = ln(*A*) − *E*_a_/*RT* (2)

where *A* represents the pre-exponential factor, *E*_a_ is the activation energy, and R is the ideal gas constant. Figure 7 shows the logarithmic values of the initial decomposition rate (ln*k*) versus the inverse temperature (1/T) for the three IHEM−1 nanoparticles at different temperatures. The fitted values are also listed in Table 3. The activation energy determined via the simulations represents the apparent activation energy of the decomposition reaction. The obtained activation energies (34–44 kJ·mol^−1^) are significantly lower than those of bulk explosives (211 kJ·mol^−1^) [47] and the experimental values of IHEM−1 (220.3 and 219 kJ·mol^−1^) [1]. This indicates that nanocrystal explosives have significantly high sensitivity, which is also proven in the experimental results [39,40,41,42,43].

## 3. Computational Methods

**Figure 8:**
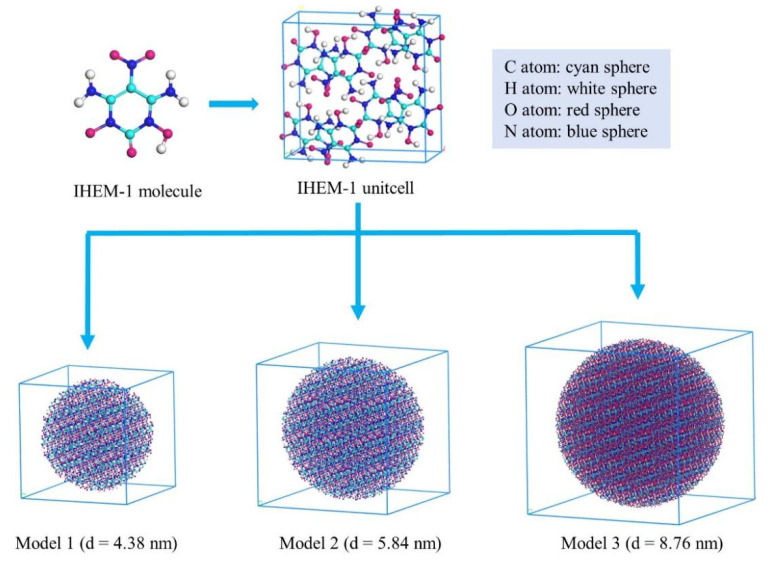
Schematic of molecular structure, unit cell structure and three nanoparticles of IHEM−1.

All molecular dynamics simulations were conducted using the ReaxFF-lg reactive force field [18], as implemented in the LAMMPS software package (Version: 5 Jun 2019) [48]. OVITO software package (Version: 3.6.0) [49] was employed for crystal visualization. The initial crystal structure of IHEM−1 was taken from the Cambridge Crystallographic Data Centre (CCDC code: 2062676). IHEM−1 possesses an orthorhombic P*cab* space group containing eight molecules in a unit cell with lattice vectors of a = 14.16278 Å, b =13.8636 Å, and c = 6.8291 Å. Subsequently, each unit cell was duplicated in all three directions to build a large super cell. Finally, three IHEM−1 nanoparticles were constructed with varying diameters, as shown in Figure 8. The nanoparticles were placed within the simulation box’s center. The three systems comprise 4864 atoms (Model 1, 4.38 nm), 11,476 atoms (Model 2, 5.84 nm), and 38,760 atoms (Model 3, 8.76 nm), respectively. Model 1, Model 2, and Model 3 are shortened as M1, M2, and M3, respectively. Detailed parameters of the three IHEM−1 models are shown in Table 4.

Andersen and Nosé–Hoover thermostats were used to regulate the pressure and temperature, respectively. RMD simulations were conducted over 200 ps using a time step of 0.1 fs. The trajectory files of atoms, molecular species, and their bonds were recorded every 10 fs. Heating was performed at 2000, 2500, 3000, and 3500 K for each system prior to conducting isothermal–isochoric MD (NVT MD) simulations at these temperatures with a damping constant of 10 fs. A bond order of 0.3 was used to determine the formation of chemical bonds [50]. The output files provide detailed information regarding reactants and products. Consequently, the fitting of the kinetic parameters can be achieved using the Arrhenius equation.

## 4. Conclusions

In this study, the effects of the particle’s size and temperature on the decomposition mechanisms of the IHEM−1 nanoparticles were simulated using the ReaxFF-lg molecular dynamics method. The results show that the initial decomposition pathways of the IHEM−1 molecules are similar for the nanoparticles with different sizes at different temperatures. The bimolecular polymerization reaction is the first step in the decomposition process. There are some differences in the quantity of the species generated during decomposition and its evolution trends, while the size of the nanoparticle does not affect the initial decomposition pathway. The formation of the hydroxyl radicals is the main decomposition mechanism with the highest reaction frequency. The degradation rate of the IHEM−1 molecules gradually rises with the increasing temperature. The IHEM−1 nanoparticles with smaller sizes exhibit greater decomposition rate constants. The activation energies for the decomposition are lower than the reported experimental values of bulk explosives, which suggests a higher sensitivity. Our study uncovers the nano effects on the thermal decomposition of nano-explosives, shedding light on the crucial facets of the decomposition process. These results could provide a valuable reference for the design of new nano-explosives.

## Figures and Tables

**Table 1:** Decay time (t, ps) to zero of the reactants in the decomposition of the three models at different temperatures.

T (K)	Model 1	Model 2	Model 3
2000	3.54	4.96	7.785
2500	1.885	2.56	3.765
3000	1.255	1.485	1.915
3500	0.745	1.15	1.195

**Table 2:** Detailed initial reaction pathways in the decomposition of the three models at different temperatures. The cyan, white, blue and red spheres stand for C, H, N, and O atoms, respectively.

Path No.	Type	Reactant	Product
Path 1	Intramolecular H transfer	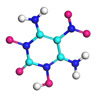	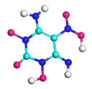
Path 2	Intramolecular H transfer	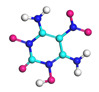	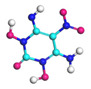
Path 3	Intermolecular H transfer	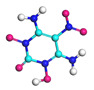	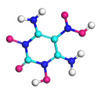
Path 4	Intermolecular H transfer	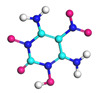	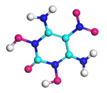
Path 5	Small moleculeformation (OH)	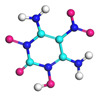	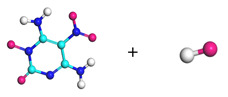
Path 6	Small moleculeFormation (NH_2_)	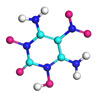	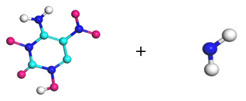
Path 7	Small moleculeformation (NO_2_)	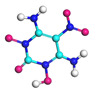	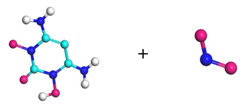
Path 8	Small moleculeformation (NH_2_)	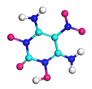	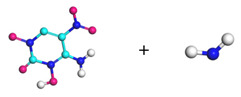
Path 9	Small moleculeformation (H)	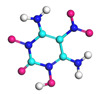	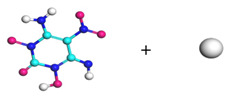
Path 10	Small moleculeformation (H)	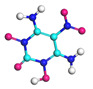	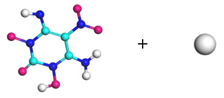
Path 11	Small moleculeformation (H_2_O)	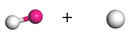	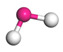
Path 12	Ring breakage (C-N)	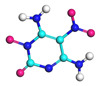	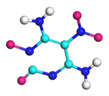
Path 13	Ring breakage (C-N)	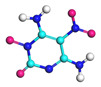	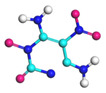
Path 14	Ring breakage (C-N)	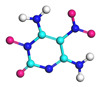	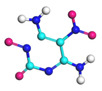
Path 15	Ring breakage (C-N)	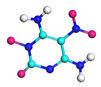	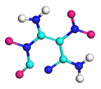
Path 16	Rearrangement of –NO_2_	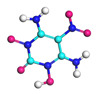	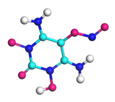
Path 17	Bimolecular polymerization	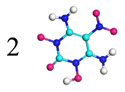	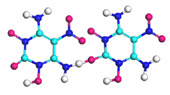

**Table 3:** Activation energies (*E*_a_, kJ·mol^−1^), pre-exponential factors (lnA, ps^−1^), and rate constants (*k*, ps^−1^) for the decomposition reactions of the three models at different temperatures.

T (K)	*k*_M1_/ps^−1^	*k*_M2_/ps^−1^	*k*_M3_/ps^−1^
2100	4.44	3.52	2.70
2400	6.92	5.95	4.26
2700	9.66	7.91	6.11
3000	12.51	8.36	8.27
*E*_a_ (kJ·mol^−1^)	40.03	34.81	43.06
ln*A* (ps^−1^)	3.88	3.40	3.56

**Table 4:** Detailed parameters of the three IHEM−1 models.

Model	Atoms	Molecules	Length of Box (Å)	Particle Diameter (nm)
1	4864	256	43.883	4.38
2	11,476	604	58.511	5.84
3	38,760	2040	87.767	8.76

## Data Availability

Data are contained within the article.
